# Bacteriophage infections of microbiota can lead to leaky gut in an experimental rodent model

**DOI:** 10.1186/s13099-016-0109-1

**Published:** 2016-06-23

**Authors:** George Tetz, Victor Tetz

**Affiliations:** Human Microbiology Institute, 303 5th Avenue, Suite 2012, New York, NY 10016 USA

**Keywords:** Bacteriophage, Intestinal permeability, Microbiota

## Abstract

Increased intestinal permeability and translocation of gut microbiota from the intestinal lumen to the systemic circulation predispose patients to various diseases and may be one of the main triggers thereof. The role of microbiota in increased intestinal permeability is under intensive investigation. Here, we studied alterations in the host and increased intestinal permeability as a direct effect of treatment with a bacteriophage cocktail. After 10 days of challenge, the rats showed weight loss, messy hair, and decreased activity. Additionally, they displayed a significantly elevated lactulose:mannitol ratio and the level of circulating immune complexes. To our knowledge, this study demonstrates for the first time that increased intestinal permeability may be induced by bacteriophages that affect the microbiota.

**Electronic supplementary material:**

The online version of this article (doi:10.1186/s13099-016-0109-1) contains supplementary material, which is available to authorized users.

## Background

The human microbiota is comprised of bacteria, fungi, and viruses including bacteriophages, and is a very complex ecosystem that is in dynamic stability with each of its components and the host organism. Gut microbes colonize the majority of mucosal surfaces, and they play a primary role in host metabolism and are substantially involved in the development and normal action of the immune and neural systems, gastro-intestinal tract, and mucosal permeability [1, 2]. The intestinal mucosa is a complex and extremely dynamic structure that readily adapts to a variety of signals that regulate intestinal permeability [3].

Intestinal barrier dysfunction or disruption, known as “leaky gut” syndrome, is characterized by the translocation of macromolecules, bacteria or their toxins to the lamina propria, which is implicated in the pathogenesis of numerous diseases [4]. Abnormally permeable mucosal barrier is associated with various pathologies including inflammatory bowel disease, Crohn’s disease, neurodegenerative diseases, diabetes type 1, some types of cancers, cardiovascular disorders, rheumatoid arthritis, etc. [5–7]. It is currently known that the permeability of the mucosal barrier is regulated and influenced by numerous factors including the gut microbiota. Bacteria directly or indirectly modulate all components of intestinal permeability such as mucus integrity and trans- and paracellular transport [8].

Here, we evaluated the effect of primary lesions of the microbiota on intestinal permeability. To cause primary lesions of the microbiota, we used bacteriophages, which are believed to selectively interact with bacteria and to not affect eukaryotic cells [9, 10]. The objective of this study was to assess the effect of microbiota treatment with bacteriophages on the intestinal permeability in vivo.

## Methods

Group of five, healthy adult, male, albino Wistar rats were used. All experiments were performed in accordance with the guide for the care and use of laboratory animals.

Ethical approval was granted by the Human Microbiology Institute Ethics Committee (T-Ph2015). In accordance with ethical approval, consent to use human biological material was assumed following completion of consent forms.

The commercial *Salmonella* bacteriophage phage cocktail (Microgen, Russia) contains phages directed against *Salmonella enterica* serotypes: Paratyphi, Typhimurium, Heidelberg, Newport, Choleraesuis, Oranienburg, Infans, Dublin, Enteritidis, Anatum, and Newlands. Pyobacteriophage Polyvalent, another commercial phage cocktail (Microgen, Russia), contains phages directed against six pathogens: *Staphylococcus aureus*, *Streptococcus pyogenes*, *Proteus mirabilis* and *P. vulgaris*, *Pseudomonas aeruginosa*, *Klebsiella pneumoniae*, and *Escherichia coli* [36]. Phage cocktail (1.5 ml [1 × 10^6^ plaque-forming units/ml] of each phage according to the manufacturer’s instruction) was given daily to animals (n = 5) for 10 days. Each animal before bacteriophage challenge was used as its own control.

The lactulose:mannitol ratio was measured as a marker of intestinal permeability 2 days before and 10 days after phage challenge as described by Meddings et al. [11]. Lactulose (L7877, Sigma-Aldrich) and mannitol (M8429, Sigma-Aldrich) were utilized for all arms of the study. For permeability testing of both probes simultaneously, animals were fasted for 4 h and then gavaged with 120 mg lactulose and 80 mg mannitol in 2 ml of water. Animals were placed in metabolic cages, and the urine passed over 24 h after the gavage was collected and assayed for the concentration of each probe by gas chromatography as described previously [12].

We measured serum circulating immune complexes (CIC) to evaluate possible systemic inflammatory response to the alterations caused by bacteriophage infection. Heparinized blood samples were collected at day 0 and day 10 from the tail vein of rats under sterile conditions. CIC were evaluated by sedimentation with a 4.0 % polyethylene glycol solution followed by spectrophotometry as described by Ramos et al. [13]. The CIC concentration was evaluated as the difference between the values of the probes before and after bacteriophage challenge.

All results are reported as the mean ± standard error (SE). Non-parametric paired Wilcoxon signed rank test was applied to analyze pre- and post-challenge differences. *P* < 0.05 was considered significant.

## Results and discussion

This study demonstrated that increased intestinal permeability may be induced by bacteriophages that affect the microbiota. We have previously confirmed the functionality and selectivity of bacteriophage cocktails (unpublished data). Ten days of administration of a bacteriophage cocktail active against *Staphylococcus* spp., *Streptococcus* spp., *Proteus* spp., *Pseudomonas* spp., *E. coli*, *K. pneumonia*, and *Salmonella* spp. did not lead to apparent clinical changes in the gastrointestinal tract or abnormal stool in rats. At the same time, all animals showed weight loss, messy hair, and decreased activity starting from the fifth day of the bacteriophage treatment, which are considered to be related to the translocation of endotoxins across leaky mucosa.

**Fig. 1:**
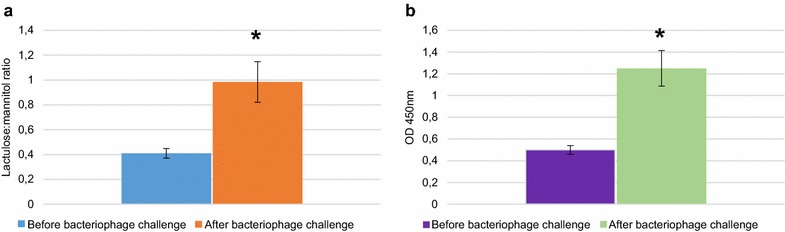
Disruption of intestinal barrier integrity in rats treated with bacteriophage cocktail. **a** Changes in lactulose:mannitol ratio before and after treatment with the bacteriophage cocktail; **b** Serum concentrations of CIC before and after treatment with the bacteriophage cocktail. Data are expressed as the mean ± SE. *P < 0.05 (Wilcoxon signed-rank test). Data of the changes in lactulose and mannitol excretions after bacteriophage challenge in each animal are presented in Additional file 1: Table S1

We performed a lactulose-mannitol permeability test to determine whether bacteriophages may cause microbiota diseases resulting in alterations in the host organism in the form of increased intestinal permeability [11]. Thus, we compared alterations of urinary mannitol and lactulose excretion and changes in the lactulose:mannitol ratio 2 days before and on the 10th day after daily challenge with the bacteriophage cocktail. The excretion of mannitol was slightly but not significantly reduced after bacteriophage challenge as compared to before. At the same time, rats exhibited a significant increase (*P* < 0,05) in lactulose excretion after bacteriophage treatment as compared to before treatment. The animals displayed a significantly elevated lactulose:mannitol ratio (*P* < 0.05), which was considered to reflect increased intestinal permeability (Fig. 1; Additional file 1: Table S1). The increase was at least 2,4-fold in all animals. All animals had a leaky gut with a benchmark lactulose/mannitol ratio >0.46 [12].

Next, we measured the serum concentrations of CIC to determine whether alterations caused by bacteriophages induced a systemic inflammatory reaction in the rats. The presence of CIC is an element of the normal immune response, and elevated levels of CIC are associated with different pathological conditions including intoxication [14]. As shown in Fig. 1, at the 10th day of treatment, the mean level of CIC was 2.5 times was higher than before treatment (*P* < 0.05), indicating endogenous intoxication, most likely caused by increased intestinal permeability and ongoing leaky gut. Untreated negative control animals that were kept under the same conditions as the treated animals did not show any changes over the study period (data not shown).

To our knowledge, this is the first study to indicate that experimental bacteriophage infection may be harmful for macro-organisms [15, 16]. The pathological effect was revealed as increased intestinal permeability, and was a result of the phages’ selective effect on the microbiota, without any direct effect on the host eukaryotic cells [17]. Over the last decade, the knowledge on abnormal intestinal permeability or leaky gut and increased bacterial translocation from the intestinal lumen has greatly progressed. This revealed the role of bacterial translocation as one of the main triggers in various poly-etiological diseases associated with chronic inflammation such as inflammatory bowel disease, Crohn’s disease, Alzheimer’s diseases, autism, diabetes, colonic neoplasia, heart failure, arrhythmias, rheumatoid arthritis, etc. [2, 5–7]. Our findings indicate that bacteriophages, which were previously not considered mammalian or human pathogens, can promote microbiota diseases and thus indirectly cause pathological conditions of mammals that are associated with leaky gut. These pathologies are believed not to be contagious or, at least, there currently is no reliable proof-of-concept of them being contagious [18, 19]. However, taking into consideration their possible association with leaky gut, it can be assumed that their incidence and distribution may be caused by phages originating from the outer environment, because bacteriophages are widely spread and humans are constantly exposed to them [20]. Finally, infection of the microbiota by bacteriophages can be considered a new group of viral diseases of mammals. Additional studies should be conducted, including those using germ-free rats as controls to confirm the effect of the bacteriophage on the gut microbiota and employing metagenomic analyses of the changes in the gut microbial communities due to the bacteriophages, leading to a leaky gut.

Further studies will be necessary to unravel microbiota diseases and to evaluate their underlying mechanisms and roles in different human pathologies.

## Additional file

10.1186/s13099-016-0109-1 Changes in lactulose and mannitol excretions after bacteriophage challenge.

## Abbreviation

CIC: circulating immune complexes
