# Supplementary material for: Bacteriophage infections of microbiota can lead to leaky gut in an experimental rodent model
Source: Gut Pathog. 2016 Jun 23;8:33. doi: 10.1186/s13099-016-0109-1 (PMC4918031; doi:10.1186/s13099-016-0109-1)
Supplement: Supplementary file 1 — 10.1186/s13099-016-0109-1 Changes in lactulose and mannitol excretions after bacteriophage challenge. [file 13099_2016_109_MOESM1_ESM.docx]

**Table 1. Changes in lactulose and mannitol excretions after bacteriophage challenge**

| **Experimental animals** | **Lactulose (pmol)** | | **Mannitol (pmol)** | |
| --- | --- | --- | --- | --- |
|  | **Before bacteriophage challenge** | **After bacteriophage challenge** | **Before bacteriophage challenge** | **After bacteriophage challenge** |
| 1 | 56 | 145 | 145 | 158 |
| 2 | 62 | 183 | 156 | 175 |
| 3 | 59 | 189 | 128 | 152 |
| 4 | 48 | 166 | 108 | 180 |
| 5 | 43 | 133 | 117 | 164 |
| **Mean** | **53.6 ± 7.9** | **163.2 ± 24.0** | **130.9 ± 19.7** | **165.8 ± 11.6** |
